# Di-Ethylhexylphthalate (DEHP) Modulates Cell Invasion, Migration and Anchorage Independent Growth through Targeting S100P in LN-229 Glioblastoma Cells

**DOI:** 10.3390/ijerph110505006

**Published:** 2014-05-09

**Authors:** Jennifer Nicole Sims, Barbara Graham, Maricica Pacurari, Sophia S. Leggett, Paul B. Tchounwou, Kenneth Ndebele

**Affiliations:** 1 Laboratory of Cancer Immunology Target Identification and Validation, Department of Biology, College of Science, Engineering and Technology, Jackson State University, 1400 J. R. Lynch Street, Jackson, MS 39217, USA; E-Mails: jennifer.n.sims@students.jsums.edu (J.N.S.); barbara.e.graham@jsums.edu (B.G.); 2 College of Science, Engineering and Technology, Jackson State University, 1400 J. R. Lynch Street, Jackson, MS 39217, USA; E-Mails: maricica.pacurari@jsums.edu (M.P.); paul.b.tchounwou@jsums.edu (P.B.T.); 3 Department of Behavioral and Environmental Health, Jackson State University, 1400 J. R. Lynch Street, Jackson, MS 39217, USA; E-Mail: sophia.s.leggett@jsums.edu

**Keywords:** glioblastoma, S100P, DEHP, RNA interference

## Abstract

Glioblastoma multiforme (GBM) is the most aggressive brain cancer with a median survival of 1–2 years. The treatment of GBM includes surgical resection, radiation and chemotherapy, which minimally extends survival. This poor prognosis necessitates the identification of novel molecular targets associated with glioblastoma. S100P is associated with drug resistance, metastasis, and poor clinical outcomes in many malignancies. The functional role of S100P in glioblastoma has not been fully investigated. In this study, we examined the role of S100P mediating the effects of the environmental contaminant, DEHP, in glioblastoma cells (LN-229) by assessing cell proliferation, apoptosis, anchorage independent growth, cell migration and invasion following DEHP exposure. Silencing S100P and DEHP treatment inhibited LN-229 glioblastoma cell proliferation and induced apoptosis. Anchorage independent growth study revealed significantly decreased colony formation in shS100P cells. We also observed reduced cell migration in cells treated with DEHP following S100P knockdown. Similar results were observed in spheroid formation and expansion. This study is the first to demonstrate the effects of DEHP on glioblastoma cells, and implicates S100P as a potential therapeutic target that may be useful as a drug response biomarker.

## 1. Introduction

Glioblastoma multiforme (GBM) is the most common and most malignant human brain tumor, with an incidence of 4 out of 100,000 per year [1]. GBM is extremely invasive and difficult to treat surgically. It is distinguished by severe and aberrant vascularization and high resistance to radiotherapy (RT) and chemotherapy [1,2]. The median survival of patients with GBM is about 1 year and only approximately 5% of patients survive no longer than 3 years [1,3]. There are no known risk factors directly related to GBM. However, long-term exposure to various carcinogens, including heavy metals and aromatic chemicals has been associated with an increased risk for GBM [4]. Exposure to these environmental chemicals can either stimulate cell proliferation or lead to cell death, therefore impacting tumor growth [5]. Phthalates are a group of industrial chemicals that are used as plasticizers, solvents, lubricants, fixatives, and as detergents in personal care products [6]. Therefore, phthalates can be identified in many widely used industrial consumer products [7]. The most well-known phthalate is di-ethylhexylphthalate (DEHP). DEHP is a principal component in polyvinyl chloride products frequently found in medical devices [8] and has the ability to penetrate the blood-brain-barrier.

It is essential to develop novel therapeutic approaches and identify therapeutic targets for advancements in glioblastoma treatment. S100P has been identified as a potential therapeutic target in the treatment of GBM. The family of S100 proteins is comprised of small dimeric constituents of the EF-hand super family of Calcium-binding proteins. Becker *et al.* purified and characterized S100P from the placenta [9]. S100P is a 95-amino acid protein and the gene coding S100P is mapped on the human chromosome 4, at 4p16 [10]. This particular chromosomal location has been associated with Huntington disease [11], Wolf–Hirschhorn syndrome [12,13], Familial Wolfram syndrome [12,14], Crohn’s disease [15] and cervical cancer [16]. S100P has been shown to aid in cancer progression through its roles in cell proliferation, survival, angiogenesis, and metastasis [17]. S100P was absent in normal breast tissue but detected in both typical and atypical hyperplasia as well as *in situ* and invasive carcinoma [18]. Therefore, S100P is highly correlated with tumor progression in breast cancer. S100P expression has also been identified in flat adenomas in the colon [19]. Moreover, S100P is specifically expressed in cancerous colon tissue [20], but not in normal colon tissue [21].

The role of S100P in glioblastoma progression has not yet been investigated. In this study, we examined whether DEHP-induced cell transformation in glioblastoma is mediated through S100P.

## 2. Experimental Section

### 2.1. Reagents

Dulbecco’s Modified Eagle’s Medium (DMEM), phosphate-buffered saline (PBS), fetal bovine serum (FBS), trypsin ethylenediaminetetraacetic acid (EDTA), puromycin, glutamine, penicillin-streptomycin, and culture supplements were purchased from Gibco (Life Technologies, Palto Alto, CA, USA). Propidium iodide (PI), S100P antibody and DEHP were purchased from Sigma-Aldrich, Inc., (St. Louis, MO, USA). Cultrex^®^ 3D spheroid cell invasion assay kit was purchased from Trevigen (Gaithersburg, MD, USA). The kit included 10× spheroid formation extracellular matrix (ECM), 3D culture qualified 96 well spheroid formation plate, and invasion matrix. All other reagents and materials were purchased from Thermo Fisher Scientific (Waltham, MA, USA).

### 2.2. Cell Culture

Glioblastoma cancer cell line, LN-229, was purchased from American Type Culture Collection (Rockville, MD, USA). The cells were cultured and maintained in DMEM containing 10% fetal bovine serum, 1% penicillin/streptomycin, and 2% glutamine. LN-229 cell lines were grown in BD primaria tissue culture dishes, with dimensions of 100 × 20 mm at 37 °C with 5% CO_2_ in a humidifier incubator and carried at 2.0 × 10^6^ cells/mL, passaging two to three times weekly as needed. Cells were pelleted by centrifugation at 1,500 rpm for 6 min at 4 °C and resuspended in fresh complete media in tissue culture plates 24 h before use in experiments to avoid any confounding gene expression that might occur because of handling.

### 2.3. Lentiviral Production and Infection

Lentiviral shRNAs targeting S100P was obtained from Harvard Medical School (Boston, MA, USA). The lentivirus was packaged by co-transfection of human embryonic cells (293T) with the shRNA expression vector, VSV-G (vesicular stomatitis virus-glycoprotein), and delta-VPR (viral protein R) plasmids at the ratio of 1:0.9:0.1, using lipofectamine 2000 (Invitrogen, Carlsbad, CA, USA). Forty-eight hours after transfection, the supernatants containing lentiviral particles were harvested and titering was performed using Hela cells.

### 2.4. shS100P Infections

LN-229 cells were plated in 10 cm dishes until 80% confluence. The day of infection, media was removed and replaced with 3 mLs of complete media supplemented with polybrene (8 μg/mL) into each plate. Two hundred and fifty (250) µL of lentivirus were added in each plate and incubated for 24 h. Cells were left to recover from infection for 24 h before initiating selection with puromycin (3 µg/mL) for three days.

### 2.5. Western Blot Analysis

Western blot analysis was performed. Briefly, cells were harvested and pelleted in an microcentrifuge (1,200 g, 5 min, 4 °C), washed in 1× PBS and resuspended in a cell lysis buffer containing 20 mM Tris (pH 8.0), 0.5% (w/v) Nonidet P-40, 1 mM EDTA, 1 μg/mL leupeptin, 1 μg/mL pepstatin, 1 mM dithiothreitol, 1 mM PMSF and 0.1 M NaCl. After a 20 min incubation period at 4 °C, supernatants were clarified by centrifugation (8,000 g, 5 min, 4 °C) and their total protein concentration was determined by the Bradford and Lowry method using Bio-Rad Protein Assay reagents in a microtiter assay. Total cellular protein (40 μg) was electrophoresed on a sodium dodecyl sulphate polyacrylamide gel (SDS-PAGE) and then transferred to a polyvinylidine difluoride membrane (GE Healthcare, Little Chalfont, Buckinghamshire, England) by electroblotting overnight in 25 mM Tris (pH 8.3), 192 mM glycine, 20% (v/v) methanol, at 15 V, 100 mA, 4 °C. The membranes were blocked with 10% (w/v) electrophoresis-grade biotin-depleted non-fat dry milk (Bio-Rad Laboratories Inc., Hercules, CA, USA) in 1× PBS, rinsed in PBS, probed with monoclonal rabbit anti- S100P at a 1:250 dilution and washed 3× in PBS. The secondary antibody, anti-rabbit whole IgG, used at 1:5,000 dilution (Transduction Laboratories San Diego, CA, USA) for one hour at room temperature. The protein bands were then visualized using an enhanced chemiluminescence (ECL) detection system (GE Healthcare, Little Chalfont, Buckinghamshire, England).

### 2.6. Cell Proliferation Assay

Cell proliferation was indirectly assessed with a colorimetric, (3-(4,5-dimethylthiazol-2-yl)-5-(3-carboxymethoxyphenyl)-2-(4-sulfophenyl)-2*H*-tetrazolium) (MTS) assay obtained from Promega (Madison, WI, USA). Cells (100 µL, number = 5,000) were plated on 96 well plates (Thermo Fisher Scientific, Waltham, MA, USA) and infected with shS100P and/or treated with 2.5 μg/µL DEHP. After 24 h of incubation, DMEM medium was removed and followed by the addition of 20 µL of MTS solution to each well. The 96 well plates were placed in an incubator at 37 °C in 5% CO_2_. The absorbance of the solution was measured at 490 nm in one hour increments for three hours using a spectrophotometer (Bio-Rad Model 550; Bio-Rad Laboratories, Inc., Hercules, CA, USA).

### 2.7. Sub G1 Apoptosis Assay

Flow cytometry was performed to assess sub G1 DNA content in an entire cell population of LN-229 cells (2 × 10^5^ cells/mL) were seeded in 24-well plates and cultured for 24 h prior to treatment. Cells were then treated with 2.5 µg/µL DEHP and incubated for 24 h. Cells were harvested, washed once with 1XPBS and suspended in 400 µL propidium iodide (PI) solution (propidium iodide 50 µg/mL, 0.1% sodium citrate and 0.1% Triton-X 100). The sub G1 DNA content was determined using a Becton Dickinson Flow Cytometer (Becton-Dickinson, San Jose, CA, USA).

### 2.8. Soft Agar Assay

Anchorage independent growth was measured using a soft agar assay. Experiments were carried out on 6-well plates. This assay is comprised of two layers. The bottom layer of agar was prepared first by dissolving 3.2 g of powdered agarose in 200 mL (1.6% agarose) of double distilled water and boiling this solution for 10 min. The bottom agar consisted of 12 mL of 1.6% agarose, and 3 mL FBS. Components for the bottom agar were mixed and 2 mL was added to each well avoiding bubbles and placed in 4 ºC for 15 min to allow agar to solidify. Plates were then incubated (37 °C, 5% CO_2_) overnight. The top agar was made by mixing 2 mL 2× DMEM, 2 mL 1.0% agarose (2.0 g of agarose in 200 mL of double distilled water boiled for 10 min), 0.5 mL FBS, 0.5 mL DMEM with FBS and penicillin/streptomycin. Untreated and treated (80 µL DEHP; 2.5 µg/µL in 3 mL DMEM) cells (number = 50,000) were added to the mixture and mixed well. Two mL of the top agar mixture was added to each well containing bottom agar. Plates were placed at 4 °C for 5 min as the agar solidified. Plates were then returned to the incubator (37 °C, 5% CO_2_) for 3 weeks. Two drops of DMEM with FBS and penicillin/streptomycin were added to each plate every two to three days to keep the plates moist over the three-week incubation period. After 3 weeks, plates were then stained with p-iodonitrotetrazolium violet (Santa Cruz, Dallas, TX, USA) in DMSO. Plates were returned to 37 °C incubator overnight. Pictures were then taken.

### 2.9. Wound Scratch Assay

Cell migration was assessed utilizing the wound scratch assay. LN-229 cells were harvested and seeded (1 × 10^6^) on a six well plate and cultured for 24 h. Media was aspirated from each well and a scratch was made on the cell mononlayer with a 200 µL pipette tip. Each well was photographed to assess the scratch and thereafter treated with DEHP. The 6-well plate was incubated at 37 °C for an additional 24 h after which each well was photographed.

### 2.10. 3D Spheroid BME Cell Invasion Assay

3D Spheroid BME Cell Invasion Assay was performed on LN-229 cells. Cells were cultured at 80% confluence and then treated with DEHP and incubated at 37 °C for 24 h. Cells for each condition were harvested and resuspended in spheroid formation ECM. This mixture was comprised of 5 µL spheroid formation ECM, 15 µL of DMEM with FBS and penicillin/streptomycin. Fifty microliters of cell suspension were added per well to the 3D culture qualified 96-well spheroid formation plate and centrifuged at 200 g for 3 min at room temperature and then incubated at 37 °C for 72 h to promote spheroid formation. Fifty microliters of the invasion matrix was added to each well in the 3D culture qualified 96-well spheroid formation plate. The spheroid formation plate was centrifuged at 300 g at 4 °C for 5 min to eliminate bubbles and position spheroids within the invasion matrix towards the center of the well. The spheroid formation plate was then transferred to the incubator at 37 °C for one hour to promote gel formation. After one hour, 100 µL of DMEM with FBS and penicillin/streptomycin was added to each well. The spheroid formation plate was incubated at 37 °C for 3 to 7 days, and spheroids were photographed in each well every two days.

### 2.11. Statistical Analyses

Statistical significance was evaluated using one-way ANOVA with multiple comparisons and Student’s *t*-test for two group comparison. The data are reported as mean ± SEM and a value of *p* < 0.05 was considered statistically significant.

## 3. Results

### 3.1. Verification of S100P Knockdown in Glioblastoma

**Figure 1:**
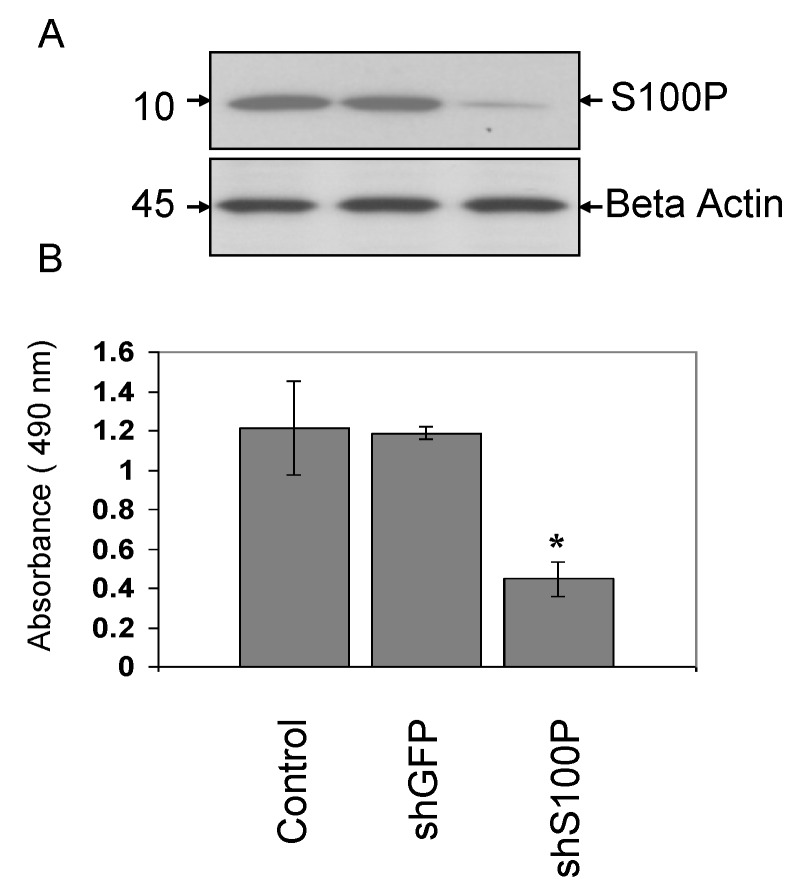
S100P knockdown decreases glioblastoma cells proliferation**. **(**A**) Western blot verification of S100P protein knockdown in LN-229 glioblastoma cells. (**B**) Cell viability was determined using MTS. The data is presented as mean ± SEM of experiments performed in triplicates. The differences were considered statistically significant with *p* < 0.05. The significant of the value is indicated by asterisks.

Notes: The differences were considered statistically significant with *p* < 0.05 as indicated by asterisk (*).

To verify S100P knockdown in LN-229 cells, western blot analysis was performed (Figure 1A). The protein expression diminished to nearly non-detectable levels in the LN-229 cells infected with shS100P, compared with those infected with the control shGFP or uninfected LN-229 cells and no changes in β-actin expression were observed.

We next asked whether shS100P knockdown is sufficient to suppress the proliferation of LN-229. Viability of LN-229 cells was measured by proliferation assay upon S100P knockdown (Figure 1B). Results showed that shS100P significantly inhibited LN-229 cells proliferation (Figure 1B). We observed similar patterns and levels of inhibition in 3 other glioblastoma cell lines (data not shown) suggesting that S100P plays a major role in proliferation of glioblastoma cells.

### 3.2. shS100P and DEHP Exposure Suppressed Glioblastoma Cell Proliferation

**Figure 2:**
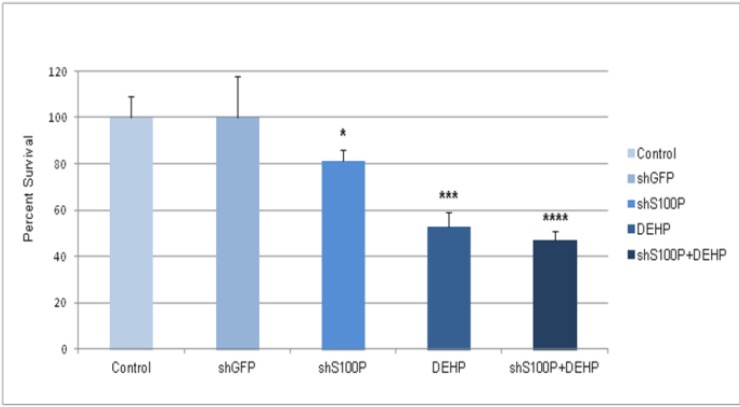
MTS assay of LN-229 cells after 48 h exposure to DEHP alone and in combination with S100P knockdown. The data is presented as mean ± SEM of experiments performed in triplicates.

Notes: The differences were considered statistically significant. One asterisk (*) indicates *p* < 0.05; three asterisks (***) indicate *p* < 0.01; four asterisks (****) indicate *p* < 0.001.

We next examined if targeting S100P regulate the effect of DEHP on cell proliferation. Treatment of glioblastoma cells with DEHP significantly inhibited cell proliferation with a 47% decrease in proliferation compared to control (Figure 2, *p* < 0.05). However, conjoint knockdown of S100P and DEHP treatment led to a greater inhibition (53%, *p* < 0.001) of LN-229 proliferation compared to either experimental group alone (Figure 2).

### 3.3. S100P Knockdown Increased Apoptosis in Glioblastoma Cells

We next sought to characterize the effect of S100P, DEHP and in combination on cell death. We chose to focus on apoptosis because it is essential for cell transformation. Propidium iodine staining was used to measure sub G1 that represents cells undergoing apoptosis. There was a significant increase in apoptosis in shS100P LN-229 cells treated with DEHP compared to the control. We did not observe an increase in apoptosis to cells treated with DEHP alone suggesting that some of the cells were dying by necrosis.

### 3.4. S100P Knockdown Reduced Anchorage Independent Growth in Glioblastoma Cells

One of the hallmarks of oncogenic transformation is the loss of anchorage independent growth as demonstrated by the ability to form colonies on soft agar. To further evaluate the role of the S100P mechanisms of action in the maintenance of the transforming phenotype of glioblastoma cells, an agar assay was used. shS100P LN-229 cells showed significant reduction in the colony formation (Figure 4). However, we did not observe any colony formation in DEHP treated cells and its combination with shS100P.

### 3.5. S100P Knockdown and DEHP Exposure Inhibited Cell Migration

The effect of S100P and DEHP on LN-229 cell migration was investigated by a scratch assay. As shown in Figure 5, cell migration was slightly inhibited in LN-229 cells infected with shS100P and significantly reduced in cells exposed to DEHP by 2.8-fold (*p* < 0.05) when compared to control (LN-229 shGFP) cells; similar findings were noted in shS100P cells treated with DEHP (*p* < 0.05).

**Figure 3:**
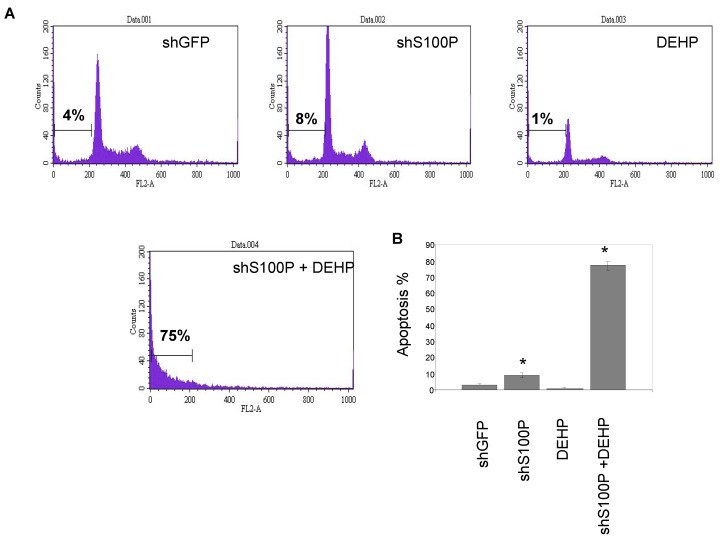
Representative apoptosis histograms of LN-229 cells exposed to DEHP alone and in combination with S100P knockdown. The percentage for apoptotic cells is shown. The changes in sub G1 DNA content were assessed by flow cytometric analysis. The experiments were repeated three times.

Note: The differences were considered statistically significant with *p* < 0.05 as indicated by asterisk (*).

**Figure 4:**
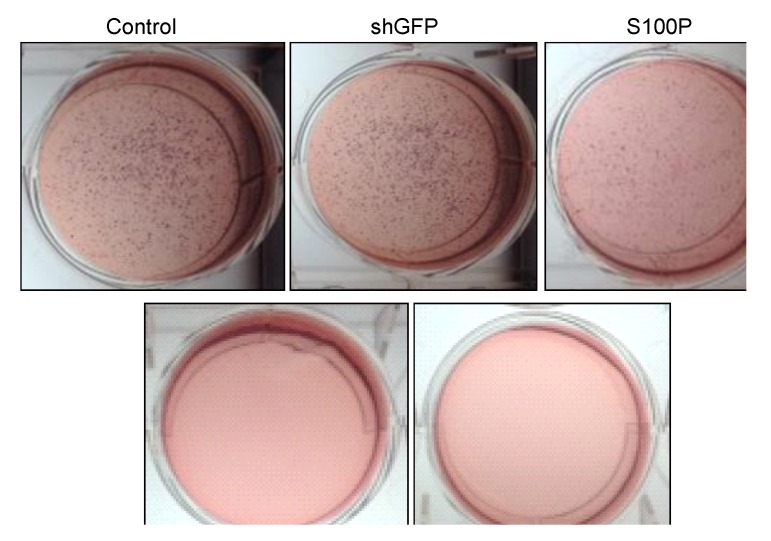
Anchorage independent growth of LN-229 cells treated with DEHP alone and in combination with S100P knockdown. Colony formation was visualized after staining the cells with p-iodonitrotetrazolium violet dye.

**Figure 5:**
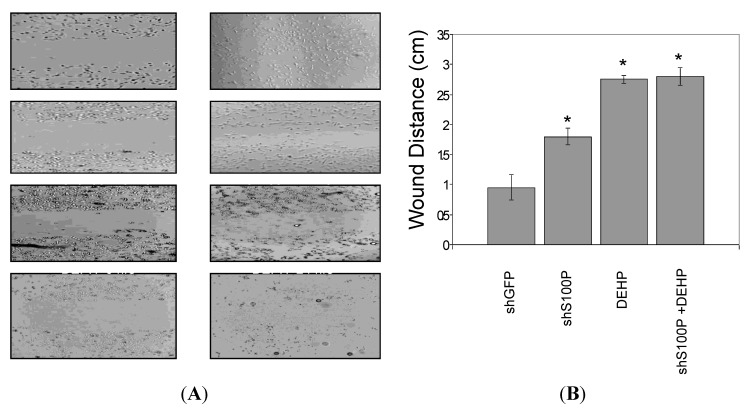
Wound healing/migration of LN-229 cells. (**A**) Representative micrographs showing LN-229 cells migration a 0 time point and after 24 h following treatment with DEHP alone and in combination with S100P knockdown. (**B**) Quantitative analysis of LN-229 cells wound distance (cm) after 24 h of treatment with DEHP alone and in combination with S100P knockdown is presented as mean ± SEM of experiments performed in triplicates.

Notes: The differences were considered statistically significant with *p* < 0.05 as indicated by asterisk (*).

### 3.6. S100P Knockdown and DEHP Exposure Reduced Spheroid Formation and Expansion

**Figure 6:**
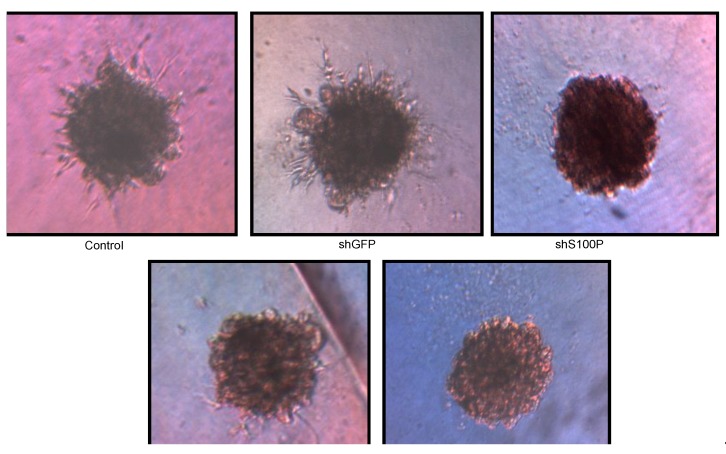
Representative micrographs of spheroid colony formation by LN-229 cells treated with DEHP alone and in combination with S100P knockdown. Microphotographs were taken after seven days of incubation.

3D spheroid BME cell invasion assay was utilized to assess the ability of LN-229 cells to invasively penetrate a barrier, containing basement membrane components, in response to chemoattractants. Control uninfected and shGFP LN-229 cells formed spheroids and showed higher spheroid expansion than cells with shS100P, DEHP, and shS100P treated with DEHP (Figure 6).

## 4. Discussion

There is an imperative need to understand the mechanisms responsible for the aggressiveness and treatment resistance of glioblastoma multiforme. This study addresses the connection between cancer and exposure to toxic DEHP substances in the environment. It is estimated that as many as two-thirds of all cancer cases are attributed to environmental causes. This environmental chemical may be present in the air, water, food, and workplace. Because of the complex interplay of many factors, it is not possible to predict whether DEHP’s environmental exposure and genetics will cause a particular person to develop cancer. Some studies in women have found that exposure to phthalates have been linked to disrupted thyroid hormone levels, increased levels of oxidative stress, and illnesses such as endometriosis and breast cancer. However, DEHP mechanisms are still not completely understood. In this study, we hypothesized that DEHP modulates cell migration, invasion and anchorage independent growth through targeting S100P in LN-229 glioblastoma cells.

S100P proteins have been found in a variety of tumors and are associated with metastasis, making them a key interest in cancer research. Overexpression of S100P has been demonstrated in several forms of cancer, including pancreas, breast, prostate, lung, and colon. However, the functional role of S100P in glioblastoma has not been elucidated. Consequently, we suppressed S100P expression in glioblastoma cancer cells using lentiviral knockdown to investigate its role in glioblastoma. Knockdown assays were performed by lentiviral infection due to its benefits over other gene therapy methods, including its ability to infect both dividing and non-dividing cells with high efficiency [22] and to achieve long-term stable expression of the transgene as well as its low immunogenicity [21]. Silencing is more specific than overexpression for determining the role of a factor in cell biology because it avoids problems associated with overexpression [23]. The expression of S100P in glioblastoma cell lines was knocked down using shS100P. In this study, we demonstrate that knockdown of S100P expression significantly inhibited cell proliferation and anchorage independent growth in LN-229 cells. A similar study of S100P knockdown in colon cancer cells also exhibited proliferation rates lower in the knockdown cells when compared to colon cancer cells expressing S100P [21]. Furthermore, in an investigation of the role of S100P in pancreatic cancer, one study revealed that cells with siRNA-silenced S100P expression grew at a significantly reduced rate compared with control siRNA-expressing cells [23]. We have shown that DEHP has the potential to cause cell transformation and it significantly inhibits cell proliferation in the presence and absence of S100P. Overexpression studies of S100P in prostate cancer reported anchorage independent growth in soft agar [24]. To determine the role of S100P on anchorage independent growth in glioblastoma cells, we examined colony formation after blocking the expression of S100P. We found that suppressing S100P expression in glioblastoma cells inhibited anchorage independent growth when compared to uninfected and shGFP cells. These results are complementary to a colon cancer study that silenced S100P by RNAi and revealed significant reductions in colony formation of cells infected with shS100P [21]. We also observed the loss of colony formation in anchorage independent growth in LN-229 cells treated with DEHP alone and in the absence of S100P.

Furthermore, we analyzed the effects of suppressing S100P expression on migration and invasion of LN-229 glioblastoma cells. Our study revealed that blocking S100P expression inhibited glioblastoma cell migration and invasion. Our results are in line with a study that silenced S100P in pancreatic cancer [23] as well as another study that silenced S100P expression in colon cancer [21] as suppressing S100P expression also reduced the rate of migration and invasion in pancreatic and colon cancer.

Exposure of DEHP on LN-229 cells caused a significant decrease in cell migration by inducing cell death. LN-229 cells were extremely sensitive to the toxic effects of DEHP. Moreover, the resultant of shS100P and DEHP exposure are not additive implicating the predominant effects of DEHP on cell migration. Van Meir *et al.* (1994) [25] showed that LN-229 glioblastoma cells have low expression levels of *p53*. Thus, we sought to determine whether S100P expression is involved in inducing apoptosis in the first growth phase of the cell cycle; our data revealed a substantial increase cell death in shS100P LN-229 cells treated with DEHP. Additionally, we demonstrated that suppressing S100P significantly inhibited spheroid expansion in LN-229 cells as compared to the control uninfected and shGFP cells. We observed that shS100Pcells treated with DEHP showed greater inhibition of spheroid expansion in LN-229; these findings were further supported by the results of a spheroid proliferation assay which revealed the same effect. We also extended the current study to examine the effect of DEHP treatment on glioblastoma cells in the presence and absence S100P expression. DEHP-induced cell death in LN-229 cells resulted in decreased cell migration.

## 5. Conclusions

To our knowledge, this is the first study to link DEHP and S100P expression in glioblastoma. Furthermore, our study revealed that silencing S100P using lentiviral mediated RNAi significantly inhibited glioblastoma cell growth and invasion. Our results indicate that S100P may contribute to the invasive nature of glioblastoma. Thus, interference with S100P may provide a novel approach for development of treatment of glioblastoma. Moreover, exposure of glioblastoma cells to DEHP revealed a significant inhibition of cell migration and invasion as well as led to a significant reduction in cell proliferation. These results taken together suggest potential effects of DEHP on human health that warrants further investigation.
